# A prospective study of the psychobehavioral factors responsible for a change from non-patient irritable bowel syndrome to IBS patient status

**DOI:** 10.1186/1751-0759-2-16

**Published:** 2008-09-25

**Authors:** Yasushi Fujii, Shinobu Nomura

**Affiliations:** 1 Faculty of Human Sciences, Waseda University, Saitama, Japan

## Abstract

**Background:**

To investigate non-patient irritable bowel syndrome (IBS) change to IBS and to determine factors predictive of the onset of IBS, individual biological factors, psychological factors, behavioral factors, and environmental factors were examined.

**Methods:**

The subjects were 105 non-patient IBS (male = 59, female = 46, average age:21.49 ± 2.37), including 68 of the diarrhea-predominant type and 37 of the constipation-predominant type selected from 1,409 university and technical college students by use of a questionnaire based on the Rome II diagnostic criteria. The subjects were followed for three years, and various characteristics and IBS symptoms were serially observed (12 times). The IBS incidence rate was calculated.

**Results:**

During the three years, 37 non-patient IBS (35.24%) changed to IBS: 28 diarrhea-predominant type and 9 constipation-predominant type. All IBS symptoms disappeared in 26 non-patient IBS subjects (24.76%). According to quantification method II (discriminant analysis), seven factors (stressor, two kinds of stress coping styles, cognitive appraisal, eating habits, sleeping time, and psychologically abuse) were adopted as a predictive model for IBS incidence and were confirmed as predictive of IBS.

**Conclusion:**

The results of this research show that non-patient IBS is a changeable state that can change into IBS or persons without symptoms. Most of the non-patient IBS subjects who became asymptomatic had had symptoms for six months or less. Furthermore, the longer a non-patient IBS subject had symptoms, the higher the risk of a change to IBS became. The findings suggest the usefulness of identifying and approaching non-patient IBS as early as possible to prevent the onset of IBS. It must be noted that the persons surveyed in the present study had only the diarrhea-predominant and constipation-predominant types. Therefore, the findings of the present study are limited only these two types. Further study including the mixed type is needed.

## Background

Irritable Bowel Syndrome (IBS) is one of the major functional gastrointestinal disorders. It is highly prevalent and is thought to be a typical psychosomatic disease. Patients with IBS do not have other functional or organic disorders such as inflammation and tumors, yet they suffer from chronic, persistent symptoms including abdominal discomfort such as pain, bloating, and altered bowel function. IBS has a good vital prognosis and is not a mortal illness. However, IBS patients usually are subjected to repeated remissions and exacerbations of the symptoms over a long period of time. Some symptoms of IBS may greatly interfere with the patients' Quality of Life (QOL) [1]. It is widely agreed that the resulting economic loss is significant [2].

Meanwhile, a significant percentage of IBS with altered abdominal symptoms that meet the Rome II diagnostic criteria is categorized as "non-patient IBS". It is estimated that "non-patient IBS" is present in about 10 – 15% of the general population, and it has been report that 75% of individuals with IBS symptoms are non-patient IBS [3]. In our survey of 921 college students, 102 (11.07%) were identified as non-patient IBS (diarrhea-predominant type 45; constipation-predominant type 38; alteration-type and uncategorized 19) [4].

It is said that a majority of the IBS patients who visit medical clinics have gone through a period of non-patient IBS. However, there are few studies that have empirically reported accurate figures for non-patient IBS; thus no body of evidence has accumulated to date. Study to clarify the disease course from healthy individual to non-patient IBS and to IBS patient and to identify the psychobehavioral factors influencing its clinical course are needed.

In this study, we did a 3-year follow-up survey of non-patient IBS using a prospective study design to determine the IBS incidence rate among non-patient IBS. In addition, we attempted to identify factors predictive of IBS onset.

Furthermore, we conducted case analyses using the expectancy value and evaluated the potential for clinical application of the factors identified as predictive of onset.

In this study, "IBS onset" was defined as the transition from non-patient IBS to treatment as an IBS patient.

## Methods

### Subjects

Screening was done of 1,409 individuals who lived in the Tokyo metropolitan area. Assessment was done by the Rome II Modular Questionnaire (RMQ) [5,6] and a questionnaire to exclude other functional and organic diseases (7 red-flag items developed by referring to the guidelines for IBS by the American Gastroenterological Association – drastic weight loss, the participant's or a family history of organic bowel disease, history of digestive surgery, blood in the stool, fever or arthralgia, abdominal pain during night sleep, and anemia). Among 136 individuals identified as non-patient IBS, a total of 105 individuals with 68 diarrhea-predominant type and 37 constipation-predominant type were selected as subjects (59 males and 46 females, mean age 21.49 ± 2.37 years).

One hundred and five of the 136 subjects met the criteria for the diarrhea-predominant type or the constipation-predominant type: The remaining 31 were not either of the above two types. According to the Rome II diagnostic criteria, subjects who do not meet the standard for either the diarrhea-predominant type or the constipation-predominant type can be included as IBS. In this study, the diarrhea-predominant type and constipation-predominant type were subject to analysis.

Rome II contains items to distinguish the mixed type in clinical practice; however the diagnostic criteria do not clearly define the mixed type as a subtype. Therefore, RMQ, the questionnaire used in this study, assumes only the diarrhea-predominant type and the constipation-predominant type, which are defined as subtypes by the diagnostic criteria (no items to identify the mixed type are included), and the subjects included in this study were selected accordingly.

We conducted a survey in which the respondents could freely describe their use of drugs to control their IBS symptom. The result of the survey shows that 95 (90.5%) of the 105 had used drugs, but we did not include the use of drugs in the statistical processing because we did not have sufficient control of the answers.

### Procedures

Each subject was asked to fill out a self-reported questionnaire, after informed consent. When obtaining consent, we informed the subjects that this study was being done to clarify the mechanism of IBS symptoms and to develop a preventive intervention method to reduce IBS symptoms. Concerning the characteristics of IBS, we explained simply and quickly, clearly specifying the diagnostic criteria. We obtained the written consent of all participants after informing them that it was being done on their own volition and that it was voluntary and that the data would not identify the individuals or be released externally.

The study period was three years over which 12 follow-up surveys were conducted at 3-month intervals. The subject did not fill in his/her name on the questionnaire due to ethical considerations, but instead was asked to set an ID number (6-digit) and use it on all surveys.

The survey items included IBS symptoms, the presence or absence of medical consultations associated with IBS symptoms in the past 3 months, and predictive factors of onset selected by literature review and meta-analysis (retrospective items were included only in the first survey). The variables used as the possible predictive factor were classified into four major groups; A. Biological factors (family history, autonomic nerve function), B. Psychological factors (stress coping, cognitive appraisal), C. Behavioral factors (life style: smoking, drinking, regularity and speed of eating, sleep duration, and exercise), and D. Environmental factors (upbringing environment and method, family function, abuse history, psychosocial stressors).

### Statistical Analysis

Quantification Method II (Discriminant analysis) was used for multivariate analysis for the identification of the factors predictive of onset, and the discrimination was done between the IBS onset group and the IBS non-onset group. The least squares method of Wilk's Lambda was used for variable selection.

Specifically, we calculated the category weight of each item selected as a candidate of the factors predictive of onset by multivariate analysis, attempting to demonstrate which factors had a large impact in the transition stage from non-patient IBS to IBS patient: IBS onset. Also, we statistically calculated the expected onset and accuracy.

### Study materials

#### IBS symptoms

Self-reported IBS Questionnaire (SIBSQ) [7,8]: IBS symptom assessment module (self-reported IBS questionnaire) developed by Fukudo et al. In this study, only stool consistency, stool frequency, and abdominal pain and discomfort frequency were used as the objective indices, and the point total of these items was considered as the IBS symptoms score.

• Stool consistency: "hard and lumpy (1 point)", "hard (2 points)", "cracked, banana-shaped (3 points)", "neither hard or soft, banana-shaped (4 points)", "soft, with some shape (5 points)", "clay-like, deformed (6 points)", "watery (7 points)"

• Stool frequency: "no defecation without laxative (1 point)", "no defecation in the last week (2 points)", "1 – 2 times in a week (3 points)", "3 – 5 times in a week (4 points)", "almost daily (5 points)", "2 – 3 times in a day (6 points)", "4 times or more in a day (7 points)"

* The above scoring is for the diarrhea-predominant type. The scoring for the constipation-predominant type is in the reverse order.

• Abdominal pain and discomfort: "absent (1 point)", "once in a week (2 points)", "2 times in a week (3 points)", "3 – 4 times in a week (4 points)", "5 – 6 times in a week (5 points)", "once daily (6 points)", "2 times or more daily (7 points)"

#### Onset predictive factors

The items selected as factors predictive of onset predictive consist of 9 indices (total of 22 lower-order indices) including family history, autonomic nerve function, stress coping, cognitive appraisal, lifestyle (smoking, drinking, regularity and speed of eating, sleep duration, and exercise), upbringing environment and method, family function, abuse history, and psychosocial stressors. The indices used in this study are listed below.

##### A. Biological factors

###### Family history

Familiar accumulation is investigated by asking the presence of IBS-diagnosed relatives within the second-degree.

###### Autonomic nerve function

Toho Medical Index (TMI) [9] is a questionnaire developed by Nakano, which consists of two factors including autonomic nerve symptoms (43 items) and psychiatric symptoms (51 items). Its question items are answered as "Yes (1 point)" or "No (0 point)". In this study, only the point total of the 43 questions concerned with autonomic nerve symptoms was used.

##### B. Psychological factors

###### Stress coping

Coping Inventory for Stressful Situation (CISS) is a questionnaire developed by Endler & Parker [10]. Its Japanese version was confirmed for reliability and validity by Furukawa et al [11]. It contains 48 items, which are assessed in a 5-grade scale from "Never did (0 point)" to "Always do (5 points)". It consists of three factors, task-oriented coping, emotion-oriented coping, and avoidance-oriented coping (16 items each).

###### Cognitive appraisal

Cognitive Appraisal Rating Scale (CARS) is a questionnaire developed by Suzuki and Sakano [12], which consists of 8 items in four factors (two items each) of "Appraisal of Effect", "Appraisal of Threat", "Commitment" and "Control probability", and that is answered in a 4-grade scale from "Don't think so (0 points)" to "Strongly think so (3 points)". The CARS was developed to measure "Cognitive Appraisal" according to the stress model by Lazarus & Folkman [13].

##### C. Behavioral factors

###### Lifestyle

From eight items of the Health Practice Index (HPI) for the judgment of lifestyle by Morimoto and Maruyama [14], lifestyle factors such as smoking, drinking and exercise were assessed by the presence of each habit and the frequency (if present), dietary habits were assessed by mealtime regularity and meal speed, and the sleep duration was assessed by average hours of sleep per day.

##### D. Environmental factors

###### Upbringing environment and method

According to a questionnaire developed by Masuda et al [15], upbringing environment was assessed by five items as follows: (1) by whom the subject was raised to 18 years of age (both parents, single parent, non-parent), (2) whether the subject was living with grandparent(s), (3) whether both parents were working, (4) whether the subject had frequently taken meals alone, and (5) whether the parents had been divorced or separated. How they were raised was assessed by 7 items as follows: (1) whether the subject had recall of being loved by parents, (2) whether the subject had recall of being hugged by parents, (3) whether the subject was strictly disciplined, (4) whether the subject had been intimidated by parent(s), (5) whether the subject had been spoiled, (6) whether the subject was given whatever he/she wanted, (7) whether the subject was praised frequently. Each was answered as "Yes (1 point)" or "No (0 point)".

###### Family function

Child-Evaluated Family Function (CEFF) is a questionnaire developed by Masuda et al [16] that consists of five items as follows: (1) if the connection between family members was strong, (2) if the subject had his/her own place in the family, (3) if the family was a safe place for the subject, (4) if the subject could be supported by the family, and (5) if the subject could talk to the family about any concerns and ask for advice. Each was answered on a 3-grade scale from "Don't think so (0 point)" to "Think so (2 points)". Judgment was made by the point total between 0 to 10 points. The higher the points, the better the family function. In this study, subjects over 18 years were asked to answer by recalling the period up to 18 years of age.

###### Abuse history

By referring to questionnaires by Masuda et al [15] and by Handa et al [17], physical abuse history (five items) and psychological abuse history (five items) were assessed on a 3-grade scale from "Did not suffer (0 point)" to "Frequently suffered (2 points)". These are questions about the period up to 18 years of age, similar to the questionnaire about family function.

###### Psychosocial stressors

Life Health Questionnaire (LHQ) was developed by Nomura [18] and has been confirmed for reliability and validity. Both life events and daily hassles were assessed for the past year.

## Results

### Changes in non-patient IBS over 3-years of follow-up

**Figure 1:**
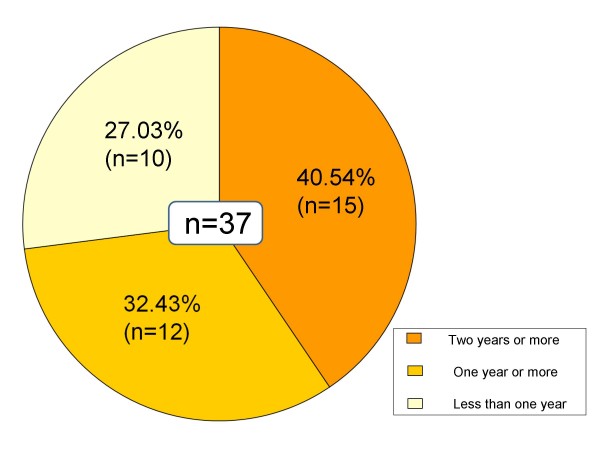
**IBS symptom retention (Changed to IBS)**. In the group that progressed to IBS, 15 cases (11 diarrhea-predominant type, 4 constipation-predominant type) had IBS symptoms for two years or more, 12 cases (10 diarrhea-predominant type, 2 constipation-predominant type) for one year or more, and 10 cases (7 diarrhea-predominant type, 3 constipation-predominant type) for less than one year.

**Figure 2:**
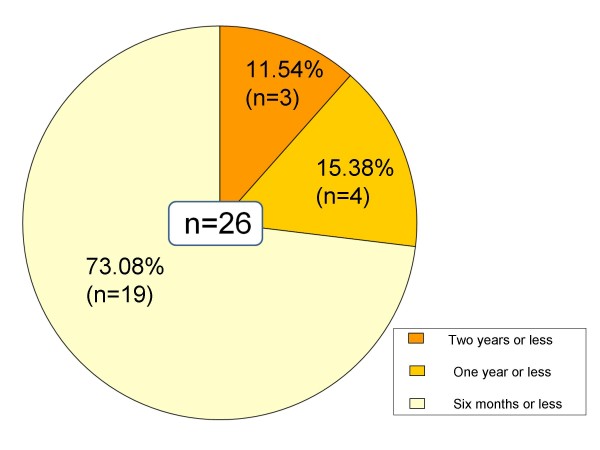
**IBS symptom retention (Changed to asymptomatic)**. All who became asymptomatic had IBS symptoms for two years or less: three (3 diarrhea-predominant type) for two years or less; four (1 diarrhea-predominant type, 3 constipation-predominant type) for one year or less; and 19 (7 diarrhea-predominant type, 12 constipation-predominant type) for six months or less.

Of 105 cases of non-patient IBS, 37 (35.24%) progressed to IBS patients during the 3-year follow-up. On the other hand, 26 cases (24.76%) became asymptomatic (Table 1). In the group that progressed to IBS, 15 cases (11 diarrhea-predominant type, 4 constipation-predominant type) had IBS symptoms for two years or more, 12 cases (10 diarrhea-predominant type, 2 constipation-predominant type) for one year or more, and 10 cases (7 diarrhea-predominant type, 3 constipation-predominant type) for less than one year (Figure 1). All who became asymptomatic had IBS symptoms for two years or less: three (3 diarrhea-predominant type) for two years or less; four (1 diarrhea-predominant type, 3 constipation-predominant type) for one year or less; and 19 (7 diarrhea-predominant type, 12 constipation-predominant type) for six months or less (Figure 2).

**Table 1:** non-patient IBS change after three years

	Became asymptomatic	Preservation of the symptoms	Progressed to IBS
Total	26(24.76%)	42	37(35.24%)
Diarrhea-predominant type	11	29	28
Constipation-predominant type	15	13	9

### Changes in IBS symptoms and factors predictive of onset just before the onset of IBS

**Figure 3:**
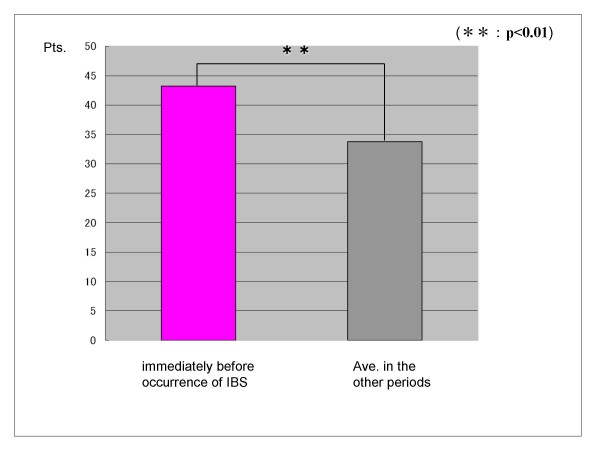
**Difference of the stressor (daily hassles) scores of the group that changed to IBS**. In the group that progressed to IBS, there was a significant increase in the stressor (daily hassles) score just before onset than earlier average scores, by t-test (p < 0.01).

**Figure 4:**
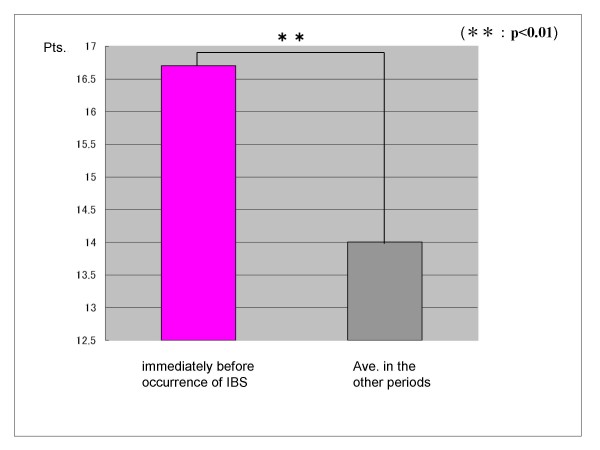
**Difference of the IBS symptoms scores of the group that changed to IBS**. In the group that progressed to IBS, there was a significant increase in the IBS symptoms score just before onset than earlier average scores, by t-test (p < 0.01).

In the group that progressed to IBS, there was a significant increase in stressor (daily hassles) score and IBS symptoms score just before onset than earlier average scores by t-test (p < 0.01) (Figure 3) (Figure 4). However, other factors predictive of onset showed no significant change just before onset.

### Factors predicting the onset of IBS

**Figure 5:**
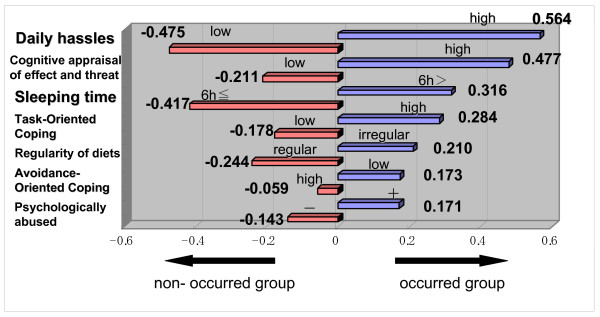
**Chart of category weight obtained from multivariate analysis for the occurrence and the non-occurrence group**. To discriminate whether or not IBS had developed, seven explanatory variables were selected. A graph of category weight depending on the quantification method II (discriminate analysis) enabled extraction of patients who have developed IBS. This figure showed each of the factors predictive of onset in order of highest influence. Using the total of these discriminate coefficients as the predictive value, we estimated that subjects with positive predictive values had high risk of developing IBS, while those with negative predictive values had low risk of developing IBS. In our subjects, the ratio of predictive value was as high as 97/105 (92.38%).

To discriminate whether or not IBS had developed, seven explanatory variables were selected. A graph of category weight depending on quantification method II (discriminate analysis) enabled extraction of patients who had developed IBS. The variables (discriminate coefficient) were as follows: (1) high stressor (daily hassles) score (0.564), (2) high cognitive appraisal score for effect and threat (0.477), (3) short sleep (6 hours or less) (0.316), (4) high task-oriented coping score (0.284), (5) low score of regularity of diet (0.210), (6) low avoidance-oriented coping score (0.173), and (7) history of being psychologically abused (0.171) (Figure 5).

Using the total of these discriminate coefficients as the predictive value, we estimated that subjects with positive predictive values had high risk of developing IBS, while those with negative predictive values had low risk of developing IBS. In our subjects, the ratio of predictive value was as high as 97/105 (92.38%).

### Review of cases using predictive value

Case 1 shown in Table 2 had a positive predictive value of 0.380, suggesting high risk of developing IBS. This person actually visited a hospital about five months after the initial examination and was diagnosed as having IBS. Case 2 shown in Table 3 had a negative predictive value of -0.226, suggesting low risk of developing IBS. The case did not develop IBS during the one-year follow-up.

**Table 2:** Case 1

Age 24, Female
**Symptoms**: Beginning in the sixth month, abdominal pain, abdominal discomfort, feeling of residual stool, and sense of abdominal fullness which are relieved with bowel movement and/or caused by changes in bowel movement.
**Stool frequency**: 4 times/day
**Stool consistency**: Watery
**Psychological problems**: Has anxiety/tension in the company with a little increase in missed workdays due to abdominal pain.
**Predictive value **= - 0.475 (Daily hassles) + 0.477 (Cognitive appraisal of effect and threat) + 0.316 (Sleeping time) - 0.178 (Task-Oriented Coping) + 0.210 (Regularity of diets) + 0.173 (Avoidance-Oriented Coping) - 0.143 (Psychologically abused) = **0.380**
◊ Positive predictive value of 0.380, suggesting high risk of developing IBS.

**Table 3:** Case 2

Age 20, Male
**Symptoms**: Beginning in the fourth month, abdominal pain, abdominal discomfort, and feeling of residual stool which are relieved with bowel movement and/or caused by changes in bowel movement.
**Stool frequency**: 5 times/day
**Stool consistency**: Watery
**Psychological problems**: Has scenes of psychological conflict at home and in school and thus has no place where the subject feels at ease. Has strong anxiety/tension.
**Predictive value **= 0.564 (Daily hassles) - 0.211 (Cognitive appraisal of effect and threat) - 0.417 (Sleeping time) + 0.284 (Task-Oriented Coping) - 0.244 (Regularity of diets) - 0.059 (Avoidance-Oriented Coping) - 0.143 (Psychologically abused) = **-0.226**
◊ Negative predictive value of -0.226, suggesting low risk of developing IBS.

### The relationship between gender or age and IBS onset

We compared the incidence rate by gender using x^2 ^test, but no significant difference was shown. In addition, reviewing gender bias of the factors predictive of onset using quantification method II (discriminant analysis) yielded similar results in the top five factors((1) high stressor (daily hassles) score, (2) high cognitive appraisal score for effect and threat, (3) short sleep (6 hours or less), (4) high task-oriented coping score, (5) low score of regularity of diet) and showed only difference of shifting the order of the rest of two factors(male: (6) low avoidance-oriented coping score, (7) history of being psychologically abused, female: (6) history of being psychologically abused, (7) low avoidance-oriented coping score).

A t-test was performed to assess the difference in age between the change to IBS group and the not changed group, which showed no significant difference. Also, for factors identified as predictive of onset, there was no effect of age (by using regression analysis).

## Discussion

The objectives of this study were to follow up subjects with non-patient IBS through a prospective study design, to survey for the incidence of IBS, and to investigate factors predictive of onset. Studies of the mechanism of IBS onset seem very important as they suggest clues to prevention of the disease.

In our study, 35.24% (n = 37) of the subjects in the non-patient IBS group (n = 105) progressed to IBS during the 3-year follow-up period. This result constitutes evidence of progression from non-patient IBS to IBS and also suggests the necessity of comparison with future studies conducted in similar design. By type of symptoms, 41.17% (n = 28) of the diarrhea-predominant type (n = 68) compared with 24.32% (n = 9) of the constipation-predominant type (n = 37), progressed to IBS, showing a higher percentage of diarrhea-predominant type subjects. This suggests that, among subjects with non-patient IBS, diarrhea-predominant type subjects may be more liable to progression to IBS.

Concerning the history of IBS symptoms, 72.97% (n = 27) of the group that progressed to IBS (n = 37) had symptoms for one year or more, while 73.08% (n = 19) of those who became asymptomatic (n = 26) had symptoms for six months or less, which suggests that a long history of IBS symptoms involves a higher risk of onset and lower chance of becoming asymptomatic, which indicates the necessity of early detection and early treatment.

There are two forms of support this study's hypothesis. We compared the period of IBS symptoms between the group that shifted to IBS patient and the asymptomatic group by t-test. The results suggests that the length of time the subjects had symptoms in the group that shifted to IBS was significantly long (p < 0.01). Comparison of the length of time the subjects in the High group (n = 18) and Low group (n = 17) (Mean ± 1SD) had IBS symptoms shows that 66.67% (n = 12) of the High group and 29.41% (n = 5) of the Low group changed to IBS. 11.11% (n = 2) of the High group and 35.29% (n = 6) of the Low group became asymptomatic and a relationship between the continuance of the symptoms and IBS onset.

In the group that progressed to IBS, psychosocial stressor (daily hassles) score and IBS symptoms score were significantly increased just before onset over earlier average scores. However, there were no significant changes in other factors predictive of onset just before onset. The result suggests that the factors that predict onset are relatively stable onset.

In this study, we reviewed a wide variety of IBS onset factors mentioned in earlier studies [e.g. [19-23]]. The results of multivariable analysis suggested that psychobehavioral factors such as stressors, stress coping, cognitive appraisal, and lifestyle play more important roles in onset than biological factors such as family history or autonomic function.

The most predictive stressor was proved to be daily hassles. Stressors are generally divided into life events and daily hassles. While life events refer to major events such as divorce, unemployment, or death of a close relative, daily hassles refer to trifle events which everybody experiences daily and frequently. Our study thus supported the theory of Lazarus RS [13] saying that "accumulation of daily hassles, rather than major changes in life, have a stronger relationship with the disease."

We agree with the role of life events working on the occurrence or worsening of IBS symptoms referred in the preceding study [24,25], but we did not see the same result in this study. The reason may be that the subjects of this study were mainly young college students, so factors like "divorce", "unemployment", or "their partner's major disease or injury" which are rated as relatively stressful life events, were not seen.

By tracking the same subjects continuously for a period of years, we may be able to assess precisely the role of life events. However, the LHQ used in this study evaluated life events with 28 items, and we found some people who experienced "decease of close relative", "own injury", or "catastrophe". Comparing the daily hassles and life events which are not referred in the preceding study, the results of this study suggest that daily hassles had more impact, a remarkable finding.

Regarding the shift from non-patient IBS to patient, we considered the contribution of the daily hassles mechanism [13] by using covariance structure analysis in another study (Fujii Y, Nomura S, unpublished data). The results of that study show stressor (daily hassles) to be enhanced by inappropriate cognitive evaluation or stress coping. The mechanism is assumed to be that it exerts a significantly bad influence to stress responses (IBS symptoms), and the stressor directly exerts a bad influence to stress responses. For this point, further empirical study is required.

While this study suggested the importance of stress models including stressors, cognitive appraisal or coping in the onset of IBS, a notable finding was that high task-oriented coping had a strong influence on the onset of IBS. This result is different from preceding study [26,27]. Task-oriented coping is coping behavior including efforts to objectively analyze the current status or to solve problems. In our study, however, the non-patient IBS group had a significantly higher task-oriented coping score than healthy people in general [4] and a combination of stressors and task-oriented coping significantly aggravated IBS symptoms or the disease-specific QOL of IBS patients or subjects with non-patient IBS [28], warranting future detailed studies.

By the results of our case study, early detection of non-patient IBS and control of factors predicting onset may help prevent IBS onset. In addition, since people with non-patient IBS already have abdominal symptoms, high motivation toward receiving preventive intervention can be expected and a psychoeducational approach to these risk factors would be effective. Further, the factors predictive of IBS onset in our study were suggested to be aggravating factors for IBS symptoms. Approaches focusing on IBS deteriorating factors may lead to prevention of protraction of the symptoms of IBS patients.

The results of this study showed little relationship between gender or age and IBS onset. A possible reason is the fact that 95.23% (n = 100) of the subjects were vocational school or college students, who did not show any significant difference in the factors predictive of onset by gender or age as typified by "difference of stressor score caused by occupational differences". When we consider this fact, selecting a population of various backgrounds (e.g. age, gender and occupation), not with similar attributes as in this study, may be necessary if we wish to review onset of IBS in more detail.

Especially in Europe and North America, health care-seeking behavior has been raised as a factor for discriminating between non-patient IBS and IBS patients. But, this study showed a significant aggravation of IBS symptoms in a non-patient IBS group at the point just before seeing a doctor. In addition, it suggested the possibility that a complex combination of inferior factors such as the psychobehavioral factors of individuals as used in our study determines the seeing doctor behavior and that specific factors have a strong influence on seeing a doctor behavior. In the future, it would be necessary to study in more detail which onset predicting factors or what combination of variables affect seeing a doctor behavior or aggravation of IBS symptoms in subjects with non-patient IBS.

Considering that IBS, once developed, is liable to become chronic, we should continue to comprehensively understand IBS from the aspect of a time axis, and to pay attention to primary through tertiary prevention. Through further accumulation of empirical studies, we hope to propose measures to prevent onset or protracted IBS and to feed them back effectively to clinical sites.

We must add that, as a limitation of the present study, the survey mainly included groups of the diarrhea-predominant and constipation-predominant types, but did not include the mixed type, which is clinically common. Because the questionnaire of this study could identify only the diarrhea-predominant type and the constipation-predominant type, it might function as a selection bias in this study. Limiting the patients surveyed may have let to limiting the outcomes of the study and should be reconsidered in the future.

## Conclusion

The results of this study showed that non-patient IBS changed to IBS in some patients and to a symptom free state in others. Most of the subjects who recovered from a non-patient IBS state to a symptom free state had symptoms for six months or less. In addition, the longer non-patient IBS had a history of the symptoms, the higher the risk of IBS incidence became. These results suggest that detection and approaching non-patient IBS in an early stage by recognizing the predictive factors for IBS is very important.

## Competing interests

The authors declare that they have no competing interests.

## Authors' contributions

YF conceptualized and designed the study, collected the data, performed analysis and interpreted the data, and drafted the manuscript. SN supervised the design of the study, the analyses of the results and the writing of the manuscript. All authors read and approved the final manuscript.

## References

[B1] Gralnek IM, Hays RD, Kilbourne A, Naliboff B, Mayer EA (2000). The impact of irritable bowel syndrome on health-related quality of life. Gastroenterology.

[B2] Sandler RS (1990). Epidemiology of irritable bowel syndrome in the United States. Gastroenterology.

[B3] Drossman DA, Thompson WG (1992). The irritable bowel syndrome: review and a graduated multicomponent treatment approach. Ann Intern Med.

[B4] Fujii Y, Nomura S (2004). The Psychological and Physiological Characteristics in Subtypes of Irritable Bowel Syndrome [abstract]. International journal of behavioral medicine.

[B5] Shinozaki M, Kanazawa M, Sagami Y, Endo Y, Hongo M, Drossman DA, Whitehead WE, Fukudo S (2006). Validation of the Japanese version of the Rome II modular questionnaire and irritable bowel syndrome severity index. J Gastroenterol.

[B6] Drossman DA, Corazziari E, Talley NJ, Thompson WG, Whitehead WE (2000). Rome II: The Functional Gastrointestinal Disorders.

[B7] Endo Y, Yoshizawa M, Fukudo S, Sasaki M, Hongo M (2000). Panic Disorder in Irritable Bowel Syndrome. Jpn J Psychosom Med.

[B8] Shinozaki M, Fukudo S, Hongo M, Shimosegawa T, Sasaki D, Matsueda K, Harasawa S, Miura S, Mine T, Kaneko H, Arakawa T, Haruma K, Torii A, Azuma T, Miwa H, Fukunaga M, Handa M, Kitamori S, Miwa T, IBS Club Japan High Prevalence of Irritable Bowel Syndrome in Medical out-patients in Japan. J Clin Gastroenterol.

[B9] Nakano K, Okonogi K (1984). Check List and Evaluation Method of subjective symptom for Identifying Autonomic Nerve Complaints. Shinshinsho shinryo Question & Answers.

[B10] Endler NS, Parker JDA (1990). Coping inventory for stressful situations (CISS): Manual. Multi-Health Systems, Inc: Tronto.

[B11] Furukawa T, Suzuki MA, Saito Y, Hamanaka T (1993). Reliability and Validity of the Japanese Version of the Coping Inventory for Stressful Situations (CISS): A Contribution to the Cross-cultural Studies of Coping. Seishin Shinkeigaku Zasshi.

[B12] Suzuki S, Sakano Y (1998). Development of a Cognitive Appraisal Rating Scale (CARS) and its Validation. Human science research (Waseda human science research).

[B13] Lazarus RS, Folkman S (1984). Stress, appraisal, and coping.

[B14] Morimoto K, Maruyama S (2001). Lifestyle and Physical and Mental Health. Jpn J Psychosom Med.

[B15] Masuda A, Hirakawa T, Yamanaka T, Shimura M, Takei M, Koga Y, Tei C (2004). The Influence of Family Function and Bringing-up Environment on the Onset of Psychosomatic and Psychosomatic-related Diseases in Adolescence. Jpn J Psychosom Med.

[B16] Masuda A, Yamanaka T, Takei M, Hirakawa T, Shimura M, Koga Y, Tei C (2004). The Influence of Family Environment on Child-Evaluated Family Function. Jpn J Psychosom Med.

[B17] Handa M, Nukina H, Ando K, Kubo C (2008). The Relationship between Physical Abuse and Anxiety and/or Depression in the Outpatients of the Psychosomatic Medicine. Biopsychosoc Med.

[B18] Nomura S (1996). A study of the reliability and Validity of a new questionnaire for stress measurement. PhD thesis.

[B19] Drossman DA (1999). Do psychosocial factors define symptom severity and patient status in irritable bowel syndrome?. Am J Med.

[B20] Howell S, Talley NJ, Quine S, Poulton R (2004). The irritable bowel syndrome has origins in the childhood socioeconomic environment. Am J Gastroenterol.

[B21] Jarrett M, Heitkemper M, Cain KC, Tuftin M, Walker EA, Bond EF, Levy RL (1998). The relationship between psychological distress and gastrointestinal symptoms in women with irritable bowel syndrome. Nurs Res.

[B22] Kanazawa M, Endo Y, Whitehead WE, Kano M, Hongo M, Fukudo S (2004). Patients and nonconsulters with irritable bowel syndrome reporting a parental history of bowel problems have more impaired psychological distress. Dig Dis Sci.

[B23] Salmon P, Skaife K, Rhodes J (2003). Abuse, dissociation, and somatization in irritable bowel syndrome: towards an explanatory model. J Behav Med.

[B24] Gwee KA, Leong YL, Graham C, McKendrick MW, Collins SM, Walters SJ, Underwood JE, Read NW (1999). The role of psychological and biological factors in postinfective gut dysfunction. Gut.

[B25] Pinto C, Lele MV, Joglekar AS, Panwar VS, Dhavale HS (2000). Stressful life-events, anxiety, depression and coping in patients of irritable bowel syndrome. J Assoc Physicians India.

[B26] Jones MP, Wessinger S, Crowell MD (2006). Coping strategies and interpersonal support in patients with irritable bowel syndrome and inflammatory bowel disease. Clin Gastroenterol Hepatol.

[B27] Crane C, Martin M (2004). Social learning, affective state and passive coping in irritable bowel syndrome and inflammatory bowel disease. Gen Hosp Psychiatry.

[B28] Fujii Y, Nomura S (2006). The Effect of Psychosocial factors and Daily habits of Irritable Bowel Syndrome Carriers on the Symptoms and Disease-specific QOL. J Psychosom Digest Dis.

